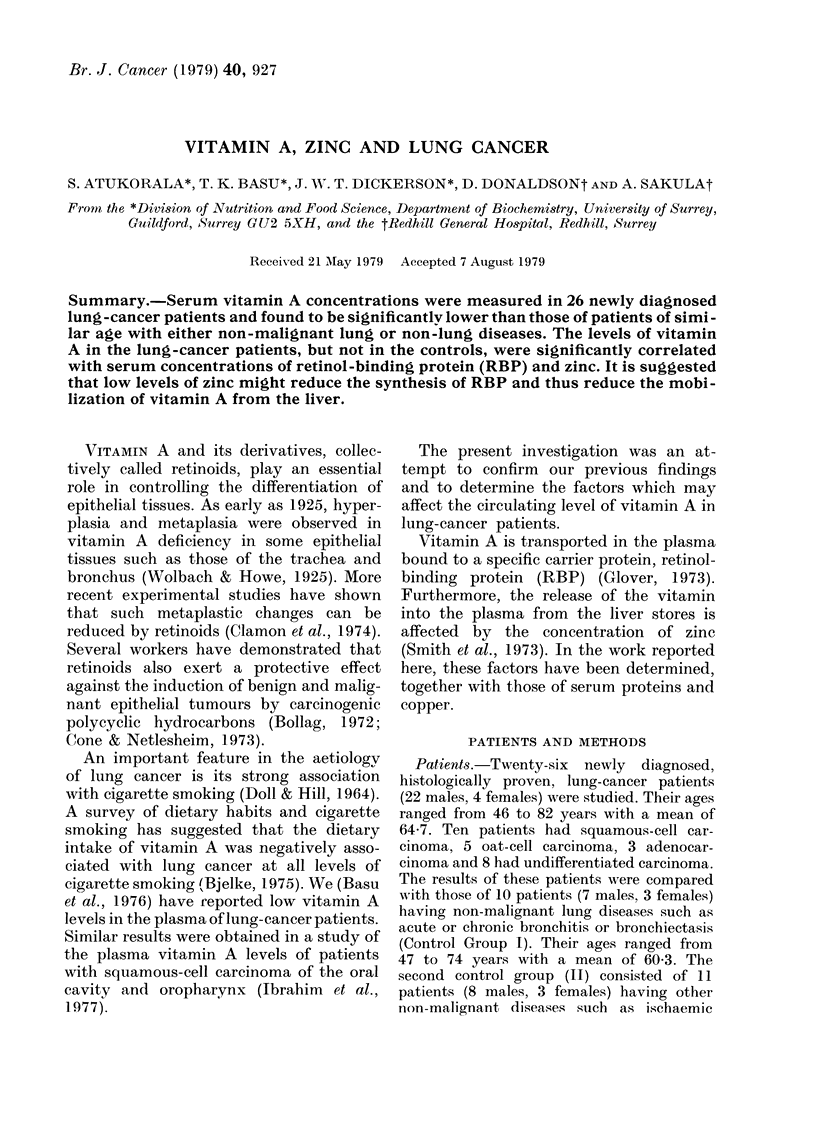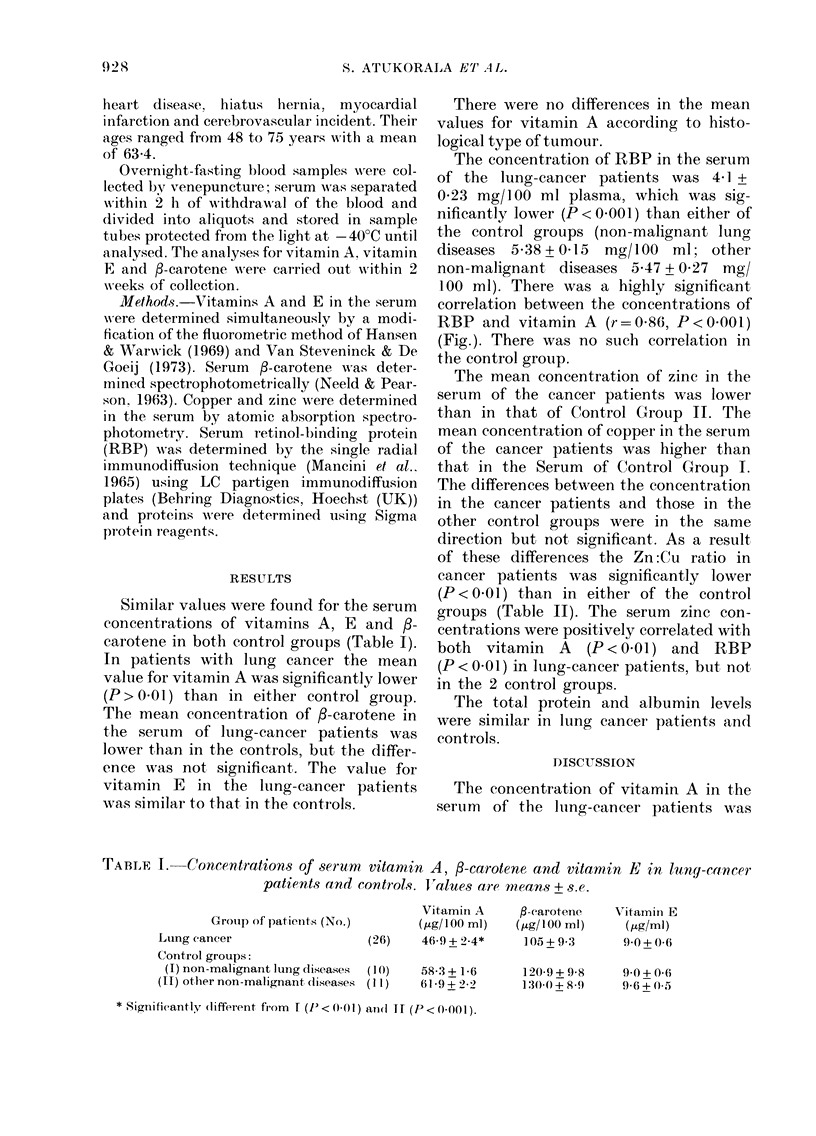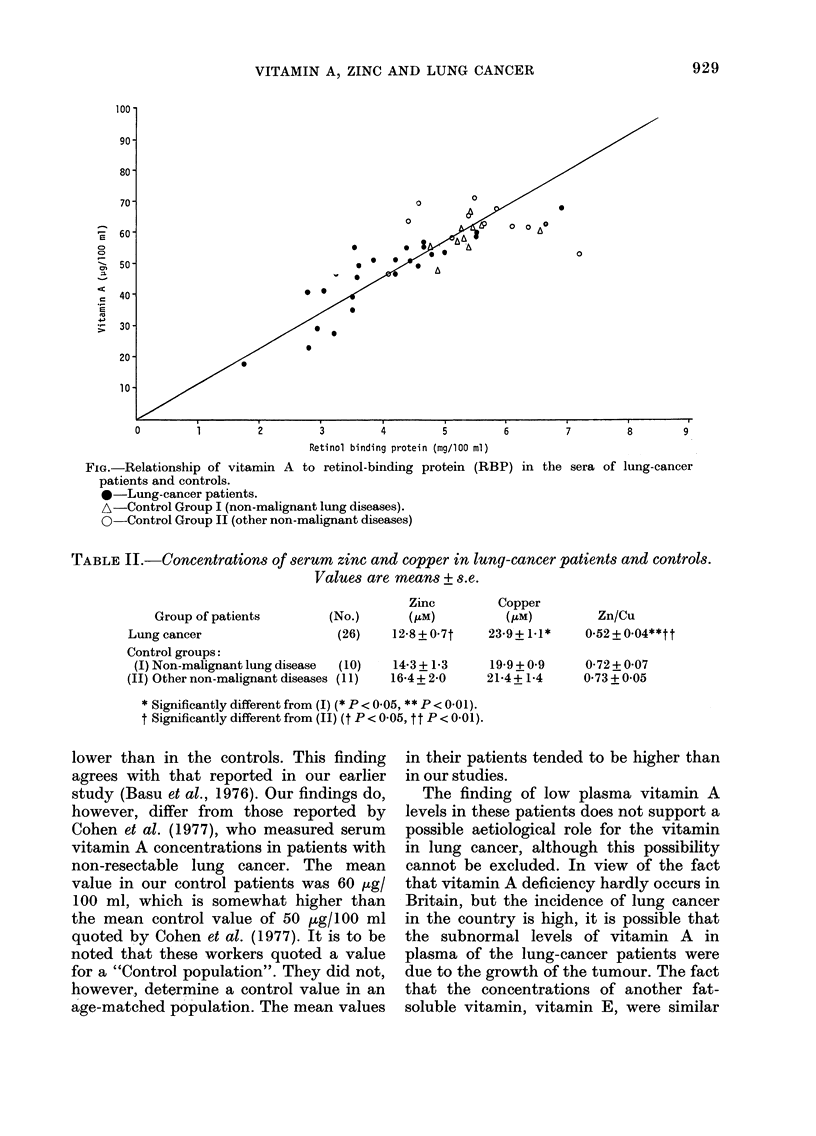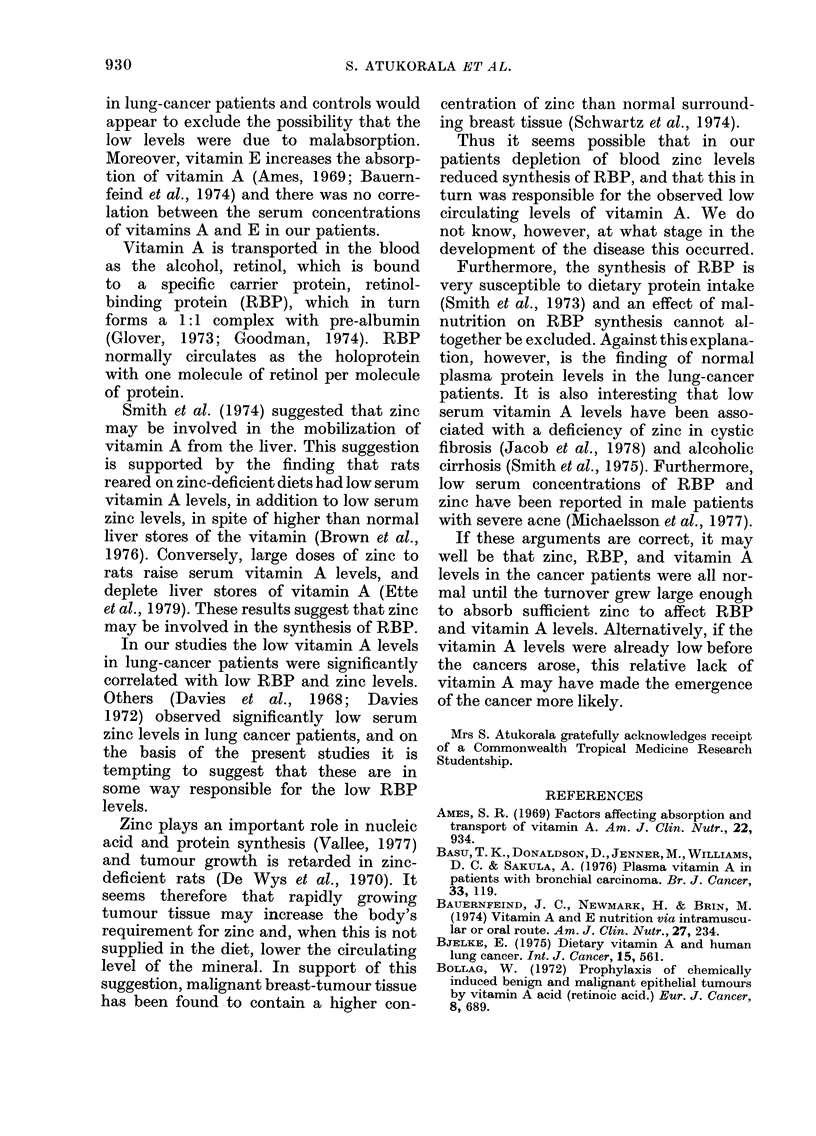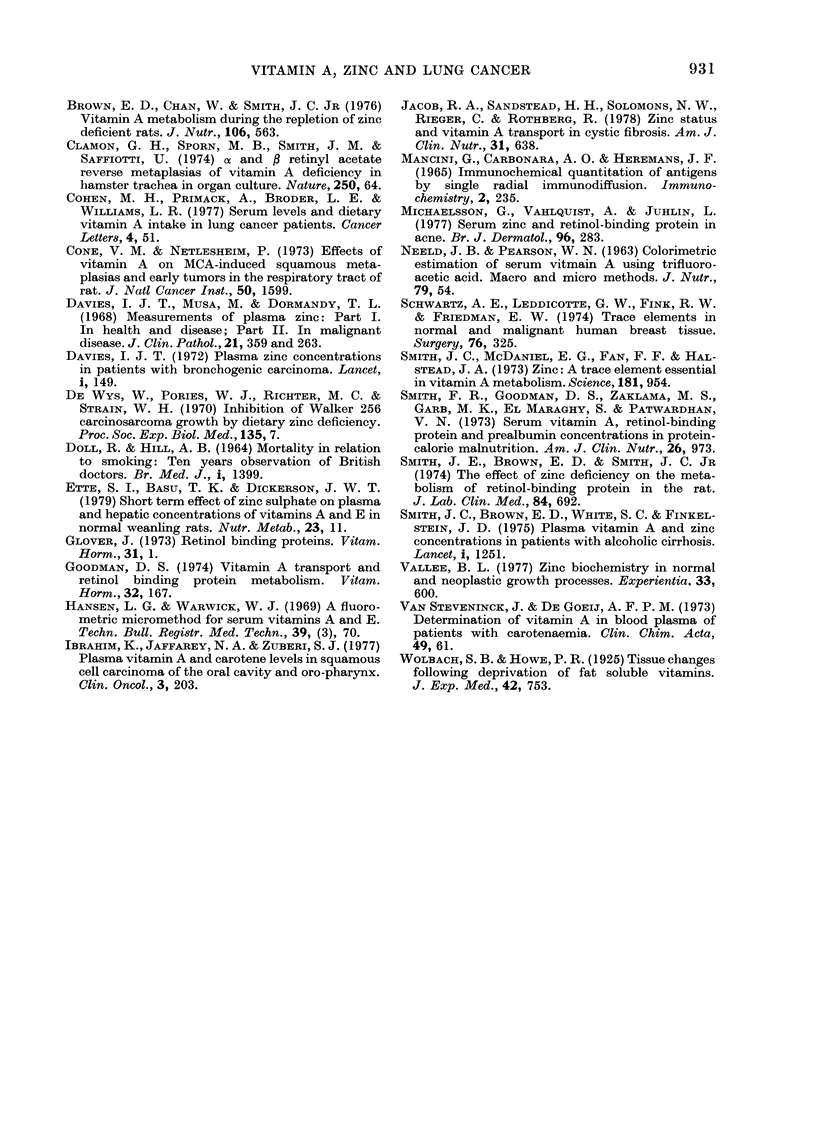# Vitamin A, zinc and lung cancer.

**DOI:** 10.1038/bjc.1979.287

**Published:** 1979-12

**Authors:** S. Atukorala, T. K. Basu, J. W. Dickerson, D. Donaldson, A. Sakula

## Abstract

Serum vitamin A concentration were measured in 26 newly diagnosed lung-cancer patients and found to be significantly lower than those of patients of similar age with either non-malignant lung or non-lung diseases. The levels of vitamin A in the lung-cancer patients, but not in the controls, were significantly correlated with serum concentrations of retinol-binding protein (RBP) and zinc. It is suggested that low levels of zinc might reduce the synthesis of RBP and thus reduce the mobilization of vitamin A from the liver.


Br. J. Cancer (1979) **40**, 927

### VITAMIN A, ZINC AND LUNG CANCER

S. ATUKORALA*, T. K. BASU*, J. W. T. DICKERSON*, D. DONALDSON† AND A. SAKULA†

*From the *Division of Nutrition and Food Science, Department of Biochemistry, University of Surrey, Guildford, Surrey GU2 5XH, and the †Redhill General Hospital, Redhill, Surrey*

Received 21 May 1979 Accepted 7 August 1979

**Summary.—Serum vitamin A concentrations were measured in 26 newly diagnosed lung-cancer patients and found to be significantly lower than those of patients of similar age with either non-malignant lung or non-lung diseases. The levels of vitamin A in the lung-cancer patients, but not in the controls, were significantly correlated with serum concentrations of retinol-binding protein (RBP) and zinc. It is suggested that low levels of zinc might reduce the synthesis of RBP and thus reduce the mobilization of vitamin A from the liver.**

VITAMIN A and its derivatives, collectively called retinoids, play an essential role in controlling the differentiation of epithelial tissues. As early as 1925, hyperplasia and metaplasia were observed in vitamin A deficiency in some epithelial tissues such as those of the trachea and bronchus (Wolbach & Howe, 1925). More recent experimental studies have shown that such metaplastic changes can be reduced by retinoids (Clamon *et al.*, 1974). Several workers have demonstrated that retinoids also exert a protective effect against the induction of benign and malignant epithelial tumours by carcinogenic polycyclic hydrocarbons (Bollag, 1972; Cone & Netlesheim, 1973).

An important feature in the aetiology of lung cancer is its strong association with cigarette smoking (Doll & Hill, 1964). A survey of dietary habits and cigarette smoking has suggested that the dietary intake of vitamin A was negatively associated with lung cancer at all levels of cigarette smoking (Bjelke, 1975). We (Basu *et al.*, 1976) have reported low vitamin A levels in the plasma of lung-cancer patients. Similar results were obtained in a study of the plasma vitamin A levels of patients with squamous-cell carcinoma of the oral cavity and oropharynx (Ibrahim *et al.*, 1977).

The present investigation was an attempt to confirm our previous findings and to determine the factors which may affect the circulating level of vitamin A in lung-cancer patients.

Vitamin A is transported in the plasma bound to a specific carrier protein, retinol-binding protein (RBP) (Glover, 1973). Furthermore, the release of the vitamin into the plasma from the liver stores is affected by the concentration of zinc (Smith *et al.*, 1973). In the work reported here, these factors have been determined, together with those of serum proteins and copper.

### PATIENTS AND METHODS

*Patients.*—Twenty-six newly diagnosed, histologically proven, lung-cancer patients (22 males, 4 females) were studied. Their ages ranged from 46 to 82 years with a mean of 64·7. Ten patients had squamous-cell carcinoma, 5 oat-cell carcinoma, 3 adenocarcinoma and 8 had undifferentiated carcinoma. The results of these patients were compared with those of 10 patients (7 males, 3 females) having non-malignant lung diseases such as acute or chronic bronchitis or bronchiectasis (Control Group I). Their ages ranged from 47 to 74 years with a mean of 60·3. The second control group (II) consisted of 11 patients (8 males, 3 females) having other non-malignant diseases such as ischaemic heart disease, hiatus hernia, myocardial infarction and cerebrovascular incident. Their ages ranged from 48 to 75 years with a mean of 63·4.

Overnight-fasting blood samples were collected by venepuncture; serum was separated within 2 h of withdrawal of the blood and divided into aliquots and stored in sample tubes protected from the light at −40°C until analysed. The analyses for vitamin A, vitamin E and β-carotene were carried out within 2 weeks of collection.

*Methods.*—Vitamins A and E in the serum were determined simultaneously by a modification of the fluorometric method of Hansen & Warwick (1969) and Van Steveninck & De Goeij (1973). Serum β-carotene was determined spectrophotometrically (Neeld & Pearson, 1963). Copper and zinc were determined in the serum by atomic absorption spectrophotometry. Serum retinol-binding protein (RBP) was determined by the single radial immunodiffusion technique (Mancini *et al.*, 1965) using LC partigen immunodiffusion plates (Behring Diagnostics, Hoechst (UK)) and proteins were determined using Sigma protein reagents.

### RESULTS

Similar values were found for the serum concentrations of vitamins A, E and β-carotene in both control groups (Table I). In patients with lung cancer the mean value for vitamin A was significantly lower ($P > 0{\cdot}01$) than in either control group. The mean concentration of β-carotene in the serum of lung-cancer patients was lower than in the controls, but the difference was not significant. The value for vitamin E in the lung-cancer patients was similar to that in the controls.

There were no differences in the mean values for vitamin A according to histological type of tumour.

The concentration of RBP in the serum of the lung-cancer patients was $4{\cdot}1 \pm 0{\cdot}23$ mg/100 ml plasma, which was significantly lower ($P < 0{\cdot}001$) than either of the control groups (non-malignant lung diseases $5{\cdot}38 \pm 0{\cdot}15$ mg/100 ml; other non-malignant diseases $5{\cdot}47 \pm 0{\cdot}27$ mg/100 ml). There was a highly significant correlation between the concentrations of RBP and vitamin A ($r = 0{\cdot}86$, $P < 0{\cdot}001$) (Fig.). There was no such correlation in the control group.

The mean concentration of zinc in the serum of the cancer patients was lower than in that of Control Group II. The mean concentration of copper in the serum of the cancer patients was higher than that in the Serum of Control Group I. The differences between the concentration in the cancer patients and those in the other control groups were in the same direction but not significant. As a result of these differences the Zn:Cu ratio in cancer patients was significantly lower ($P < 0{\cdot}01$) than in either of the control groups (Table II). The serum zinc concentrations were positively correlated with both vitamin A ($P < 0{\cdot}01$) and RBP ($P < 0{\cdot}01$) in lung-cancer patients, but not in the 2 control groups.

The total protein and albumin levels were similar in lung cancer patients and controls.

### DISCUSSION

The concentration of vitamin A in the serum of the lung-cancer patients was

TABLE I.—*Concentrations of serum vitamin A, β-carotene and vitamin E in lung-cancer patients and controls. Values are means ± s.e.*

| Group of patients (No.) | | Vitamin A (μg/100 ml) | β-carotene (μg/100 ml) | Vitamin E (μg/ml) |
|---|---|---|---|---|
| Lung cancer | (26) | 46·9 ± 2·4* | 105 ± 9·3 | 9·0 ± 0·6 |
| Control groups: | | | | |
| (I) non-malignant lung diseases | (10) | 58·3 ± 1·6 | 120·9 ± 9·8 | 9·0 ± 0·6 |
| (II) other non-malignant diseases | (11) | 61·9 ± 2·2 | 130·0 ± 8·9 | 9·6 ± 0·5 |

* Significantly different from I ($P < 0{\cdot}01$) and II ($P < 0{\cdot}001$).

FIG.—Relationship of vitamin A to retinol-binding protein (RBP) in the sera of lung-cancer patients and controls.
●—Lung-cancer patients.
△—Control Group I (non-malignant lung diseases).
○—Control Group II (other non-malignant diseases)

TABLE II.—*Concentrations of serum zinc and copper in lung-cancer patients and controls. Values are means ± s.e.*

| Group of patients | (No.) | Zinc (µM) | Copper (µM) | Zn/Cu |
|---|---|---|---|---|
| Lung cancer | (26) | 12·8 ± 0·7† | 23·9 ± 1·1* | 0·52 ± 0·04**†† |
| Control groups: | | | | |
| (I) Non-malignant lung disease | (10) | 14·3 ± 1·3 | 19·9 ± 0·9 | 0·72 ± 0·07 |
| (II) Other non-malignant diseases | (11) | 16·4 ± 2·0 | 21·4 ± 1·4 | 0·73 ± 0·05 |

* Significantly different from (I) (* $P < 0{\cdot}05$, ** $P < 0{\cdot}01$).

† Significantly different from (II) († $P < 0{\cdot}05$, †† $P < 0{\cdot}01$).

lower than in the controls. This finding agrees with that reported in our earlier study (Basu *et al.*, 1976). Our findings do, however, differ from those reported by Cohen *et al.* (1977), who measured serum vitamin A concentrations in patients with non-resectable lung cancer. The mean value in our control patients was 60 µg/100 ml, which is somewhat higher than the mean control value of 50 µg/100 ml quoted by Cohen *et al.* (1977). It is to be noted that these workers quoted a value for a "Control population". They did not, however, determine a control value in an age-matched population. The mean values in their patients tended to be higher than in our studies.

The finding of low plasma vitamin A levels in these patients does not support a possible aetiological role for the vitamin in lung cancer, although this possibility cannot be excluded. In view of the fact that vitamin A deficiency hardly occurs in Britain, but the incidence of lung cancer in the country is high, it is possible that the subnormal levels of vitamin A in plasma of the lung-cancer patients were due to the growth of the tumour. The fact that the concentrations of another fat-soluble vitamin, vitamin E, were similar in lung-cancer patients and controls would appear to exclude the possibility that the low levels were due to malabsorption. Moreover, vitamin E increases the absorption of vitamin A (Ames, 1969; Bauernfeind *et al.*, 1974) and there was no correlation between the serum concentrations of vitamins A and E in our patients.

Vitamin A is transported in the blood as the alcohol, retinol, which is bound to a specific carrier protein, retinol-binding protein (RBP), which in turn forms a 1:1 complex with pre-albumin (Glover, 1973; Goodman, 1974). RBP normally circulates as the holoprotein with one molecule of retinol per molecule of protein.

Smith *et al.* (1974) suggested that zinc may be involved in the mobilization of vitamin A from the liver. This suggestion is supported by the finding that rats reared on zinc-deficient diets had low serum vitamin A levels, in addition to low serum zinc levels, in spite of higher than normal liver stores of the vitamin (Brown *et al.*, 1976). Conversely, large doses of zinc to rats raise serum vitamin A levels, and deplete liver stores of vitamin A (Ette *et al.*, 1979). These results suggest that zinc may be involved in the synthesis of RBP.

In our studies the low vitamin A levels in lung-cancer patients were significantly correlated with low RBP and zinc levels. Others (Davies *et al.*, 1968; Davies 1972) observed significantly low serum zinc levels in lung cancer patients, and on the basis of the present studies it is tempting to suggest that these are in some way responsible for the low RBP levels.

Zinc plays an important role in nucleic acid and protein synthesis (Vallee, 1977) and tumour growth is retarded in zinc-deficient rats (De Wys *et al.*, 1970). It seems therefore that rapidly growing tumour tissue may increase the body's requirement for zinc and, when this is not supplied in the diet, lower the circulating level of the mineral. In support of this suggestion, malignant breast-tumour tissue has been found to contain a higher concentration of zinc than normal surrounding breast tissue (Schwartz *et al.*, 1974).

Thus it seems possible that in our patients depletion of blood zinc levels reduced synthesis of RBP, and that this in turn was responsible for the observed low circulating levels of vitamin A. We do not know, however, at what stage in the development of the disease this occurred.

Furthermore, the synthesis of RBP is very susceptible to dietary protein intake (Smith *et al.*, 1973) and an effect of malnutrition on RBP synthesis cannot altogether be excluded. Against this explanation, however, is the finding of normal plasma protein levels in the lung-cancer patients. It is also interesting that low serum vitamin A levels have been associated with a deficiency of zinc in cystic fibrosis (Jacob *et al.*, 1978) and alcoholic cirrhosis (Smith *et al.*, 1975). Furthermore, low serum concentrations of RBP and zinc have been reported in male patients with severe acne (Michaelsson *et al.*, 1977).

If these arguments are correct, it may well be that zinc, RBP, and vitamin A levels in the cancer patients were all normal until the turnover grew large enough to absorb sufficient zinc to affect RBP and vitamin A levels. Alternatively, if the vitamin A levels were already low before the cancers arose, this relative lack of vitamin A may have made the emergence of the cancer more likely.

Mrs S. Atukorala gratefully acknowledges receipt of a Commonwealth Tropical Medicine Research Studentship.

### REFERENCES

AMES, S. R. (1969) Factors affecting absorption and transport of vitamin A. *Am. J. Clin. Nutr.*, **22**, 934.

BASU, T. K., DONALDSON, D., JENNER, M., WILLIAMS, D. C. & SAKULA, A. (1976) Plasma vitamin A in patients with bronchial carcinoma. *Br. J. Cancer*, **33**, 119.

BAUERNFEIND, J. C., NEWMARK, H. & BRIN, M. (1974) Vitamin A and E nutrition *via* intramuscular or oral route. *Am. J. Clin. Nutr.*, **27**, 234.

BJELKE, E. (1975) Dietary vitamin A and human lung cancer. *Int. J. Cancer*, **15**, 561.

BOLLAG, W. (1972) Prophylaxis of chemically induced benign and malignant epithelial tumours by vitamin A acid (retinoic acid.) *Eur. J. Cancer*, **8**, 689.

BROWN, E. D., CHAN, W. & SMITH, J. C. JR (1976) Vitamin A metabolism during the repletion of zinc deficient rats. *J. Nutr.*, **106**, 563.

CLAMON, G. H., SPORN, M. B., SMITH, J. M. & SAFFIOTTI, U. (1974) $\alpha$ and $\beta$ retinyl acetate reverse metaplasias of vitamin A deficiency in hamster trachea in organ culture. *Nature*, **250**, 64.

COHEN, M. H., PRIMACK, A., BRODER, L. E. & WILLIAMS, L. R. (1977) Serum levels and dietary vitamin A intake in lung cancer patients. *Cancer Letters*, **4**, 51.

CONE, V. M. & NETLESHEIM, P. (1973) Effects of vitamin A on MCA-induced squamous metaplasias and early tumors in the respiratory tract of rat. *J. Natl Cancer Inst.*, **50**, 1599.

DAVIES, I. J. T., MUSA, M. & DORMANDY, T. L. (1968) Measurements of plasma zinc: Part I. In health and disease; Part II. In malignant disease. *J. Clin. Pathol.*, **21**, 359 and 263.

DAVIES, I. J. T. (1972) Plasma zinc concentrations in patients with bronchogenic carcinoma. *Lancet*, **i**, 149.

DE WYS, W., PORIES, W. J., RICHTER, M. C. & STRAIN, W. H. (1970) Inhibition of Walker 256 carcinosarcoma growth by dietary zinc deficiency. *Proc. Soc. Exp. Biol. Med.*, **135**, 7.

DOLL, R. & HILL, A. B. (1964) Mortality in relation to smoking: Ten years observation of British doctors. *Br. Med. J.*, **i**, 1399.

ETTE, S. I., BASU, T. K. & DICKERSON, J. W. T. (1979) Short term effect of zinc sulphate on plasma and hepatic concentrations of vitamins A and E in normal weanling rats. *Nutr. Metab.*, **23**, 11.

GLOVER, J. (1973) Retinol binding proteins. *Vitam. Horm.*, **31**, 1.

GOODMAN, D. S. (1974) Vitamin A transport and retinol binding protein metabolism. *Vitam. Horm.*, **32**, 167.

HANSEN, L. G. & WARWICK, W. J. (1969) A fluorometric micromethod for serum vitamins A and E. *Techn. Bull. Registr. Med. Techn.*, **39**, (3), 70.

IBRAHIM, K., JAFFAREY, N. A. & ZUBERI, S. J. (1977) Plasma vitamin A and carotene levels in squamous cell carcinoma of the oral cavity and oro-pharynx. *Clin. Oncol.*, **3**, 203.

JACOB, R. A., SANDSTEAD, H. H., SOLOMONS, N. W., RIEGER, C. & ROTHBERG, R. (1978) Zinc status and vitamin A transport in cystic fibrosis. *Am. J. Clin. Nutr.*, **31**, 638.

MANCINI, G., CARBONARA, A. O. & HEREMANS, J. F. (1965) Immunochemical quantitation of antigens by single radial immunodiffusion. *Immunochemistry*, **2**, 235.

MICHAELSSON, G., VAHLQUIST, A. & JUHLIN, L. (1977) Serum zinc and retinol-binding protein in acne. *Br. J. Dermatol.*, **96**, 283.

NEELD, J. B. & PEARSON, W. N. (1963) Colorimetric estimation of serum vitmain A using trifluoroacetic acid. Macro and micro methods. *J. Nutr.*, **79**, 54.

SCHWARTZ, A. E., LEDDICOTTE, G. W., FINK, R. W. & FRIEDMAN, E. W. (1974) Trace elements in normal and malignant human breast tissue. *Surgery*, **76**, 325.

SMITH, J. C., McDANIEL, E. G., FAN, F. F. & HALSTEAD, J. A. (1973) Zinc: A trace element essential in vitamin A metabolism. *Science*, **181**, 954.

SMITH, F. R., GOODMAN, D. S., ZAKLAMA, M. S., GARB, M. K., EL MARAGHY, S. & PATWARDHAN, V. N. (1973) Serum vitamin A, retinol-binding protein and prealbumin concentrations in protein-calorie malnutrition. *Am. J. Clin. Nutr.*, **26**, 973.

SMITH, J. E., BROWN, E. D. & SMITH, J. C. JR (1974) The effect of zinc deficiency on the metabolism of retinol-binding protein in the rat. *J. Lab. Clin. Med.*, **84**, 692.

SMITH, J. C., BROWN, E. D., WHITE, S. C. & FINKELSTEIN, J. D. (1975) Plasma vitamin A and zinc concentrations in patients with alcoholic cirrhosis. *Lancet*, **i**, 1251.

VALLEE, B. L. (1977) Zinc biochemistry in normal and neoplastic growth processes. *Experientia*, **33**, 600.

VAN STEVENINCK, J. & DE GOEIJ, A. F. P. M. (1973) Determination of vitamin A in blood plasma of patients with carotenaemia. *Clin. Chim. Acta*, **49**, 61.

WOLBACH, S. B. & HOWE, P. R. (1925) Tissue changes following deprivation of fat soluble vitamins. *J. Exp. Med.*, **42**, 753.